# Tanshinone IIA Has a Potential Therapeutic Effect on Kawasaki Disease and Suppresses Megakaryocytes in Rabbits With Immune Vasculitis

**DOI:** 10.3389/fcvm.2022.873851

**Published:** 2022-04-13

**Authors:** Hui Chen, Huiying Shu, Weiqing Su, Bo Li, Hua Zhang, Liang Li, Chao Lin, Wenfang Yi, Xiao-Yong Zhan, Chun Chen, Xiaojing Li, Yanqi Yang, Min Zhou, Mo Yang

**Affiliations:** ^1^The Seventh Affiliated Hospital, Sun Yat-sen University, Shenzhen, China; ^2^Department of Hematology and Oncology, Chengdu Women’s and Children’s Central Hospital, School of Medicine, University of Electronic Science and Technology of China, Chengdu, China; ^3^Lianjiang People’s Hospital, Zhanjiang, China; ^4^Guangdong Provincial Key Laboratory of Digestive Cancer Research, Shenzhen, China; ^5^Capital Institute of Pediatrics, Beijing, China; ^6^Sun Yat-sen Memorial Hospital, Sun Yat-sen University, Guangzhou, China

**Keywords:** tanshinone IIA, Kawasaki disease, immune vasculitis, megakaryocyte, apoptosis

## Abstract

**Background and Objective:**

It is urgent to find out an alternative therapy for Kawasaki disease (KD) since around 20% patients are resistant to intravenous immunoglobulin (IVIG) or aspirin. Tanshinone IIA is the active component of the traditional Chinese medicine Danshen (*Salvia miltiorrhiza*), which has anti-inflammatory and anti-platelet properties; however, whether or not tanshinone IIA has a therapeutic effect on KD remains unclear. Therefore, the present study aimed to examine the effect of tanshinone IIA on KD patients and rabbits with immune vasculitis, and to identify the potential mechanisms with special emphasis on megakaryopoiesis and megakaryocytic apoptosis.

**Methods:**

Kawasaki disease patients were recruited and prescribed with tanshinone IIA in the absence or presence of aspirin and IVIG, and the inflammatory responses and platelet functions were determined. Megakaryocytes (MKs) isolated from rabbits with immune vasculitis and human megakaryocytic CHRF-288-11 cells were treated with tanshinone IIA to examine the colony forming unit (CFU) and apoptosis, respectively. Microarray assay was conducted to identify potential targets of tanshinone IIA-induced apoptosis.

**Results:**

Tanshinone IIA reduced the serum levels of C-reactive protein (CRP), interleukin (IL)-1β, IL-6, and P-selectin in KD patients; such inhibitory effect was more significant compared to aspirin and IVIG. It also dose-dependently lowered the levels of tumor necrosis factor (TNF)-α and IL-8 in peripheral blood mononuclear cells isolated from KD patients. In rabbits with immune vasculitis, tanshinone IIA significantly reduced the serum levels of proinflammatory cytokines and platelet functions. In addition, tanshinone IIA significantly decreased the number of bone marrow MKs and inhibited the Colony Forming Unit-Megakaryocyte (CFU-MK) formation. In human megakaryocytic CHRF-288-11 cells, tanshinone IIA induced caspase-dependent apoptosis, probably through up-regulating TNF receptor superfamily member 9 (TNFRSF9) and the receptor (TNFRSF)-interacting serine/threonine-protein kinase 1 (RIPK1), which may contribute to its anti-platelet and anti-inflammatory properties.

**Conclusion:**

Tanshinone IIA exerts better anti-inflammatory and anti-platelet effects in treating KD patients than aspirin and IVIG. It attenuates immune vasculitis likely by inhibiting IL-mediated megakaryopoiesis and inducing TNFRSF9/RIPK1/caspase-dependent megakaryocytic apoptosis. The findings therefore suggest that tanshinone IIA may be a promising alternative therapy for the treatment of KD.

## Introduction

Kawasaki disease (KD) is a febrile disease that predominates in children under 5 years of age, with immune vasculitis as the major pathological change, especially the coronary artery damage (1). The incidence of coronary artery damage in untreated KD patients is around 25%, and it is the most common cause of acquired heart disease among children; in severe cases, it would even develop into coronary aneurysms and myocardial infarction (2). The pathogenesis of KD, however, remains incompletely understood. It is generally accepted that immune vasculitis is attributable to excessive responses of the immune system in children with genetic vulnerabilities; the abnormal T cell activation in the peripheral blood produces high concentrations of cytokines and inflammatory mediators, and results in abnormal platelet activation as well (3–6). Therefore, the conventional therapy for KD is aspirin in combination with IVIG (intravenous immunoglobulin), for their anti-inflammatory and anti-platelet effects during the acute and sub-acute phases, respectively. Unfortunately, IVIG resistance occurred in 15–25% KD patients, who are almost nine times more likely to have coronary artery injury (7–9). In addition, long-term administration of aspirin leads to gastric mucosal damage and bleeding. Thus, it is urgent to unravel the pathogenesis of KD and to find out alternative therapies.

Tanshinone IIA is the active and major lipid-soluble component of Danshen (*Salvia miltiorrhiza*), a traditional Chinese medicine, which has been used for centuries to treat cardiovascular diseases, such as angina pectoris, myocardial infarction and hypertension. In addition, being a small and lipophilic component, which can penetrate the blood brain barrier, tanshinone IIA has been reported to play a neuroprotective role (10). Furthermore, evidences also show that tanshinone IIA has anti-tumor effects by inhibiting angiogenesis and suppressing epithelial-to-mesenchymal transition (EMT) and metastasis (11, 12). The therapeutic efficacy of tanshinone IIA is attributed to its various pharmacological effects, including vasorelaxant, anti-coagulative, anti-inflammatory, immuno-regulatory, and anti-oxidant properties (13–15). Since KD is characterized by abnormal inflammatory responses and platelet activation, whether or not tanshinone IIA could be an alternative therapy for KD patients is worth further examinations.

Numerous studies have focused on the inhibitory effect of tanshinone IIA on platelet activation and aggregation, but the underlying mechanisms, especially in regard to the regulation of megakaryocytes (MK), the precursor cells that produce and release platelets, draws little attention. Megakaryopoiesis is a complex process that involves the commitment of hematopoietic stem/progenitor cells (HSC/HPCs) to MK lineage and maturation of MKs, which in turn produces platelets (16). Megakaryopoiesis and platelet production are controlled by multiple growth factors, including thrombopoietin (TPO), interleukin (IL)-1β, IL-3, IL-6, and human stem cell factor (17–19), and thereinto, the IL family is both hematopoietic and proinflammatory cytokines. Whether or not the anti-inflammatory property of tanshinone IIA affects megakaryopoiesis, and thus contributing to its anti-platelet effect remains unclear. Furthermore, the apoptosis of MKs also contributes to the balance of MK counts and functions. Signals that are activated by inflammation are involved in the regulation of apoptosis, therefore, tanshinone IIA might also play a role in controlling the apoptosis of MKs and thus the functions of platelets.

Tanshinone IIA shares similar pharmacological effects with aspirin and IVIG in regard to anti-platelet and anti-inflammation properties; however, its effect on megakaryopoiesis and apoptosis of MKs remains largely unknown. Therefore, the present study was designed to examine whether or not tanshinone IIA could be an alternative choice for the treatment of KD, and to explore the underlying mechanisms especially associated with MKs.

## Materials and Methods

### Patients With Kawasaki Disease and Healthy Controls

The study was approved by the Ethics Committee of Chengdu Women’s and Children’s Central Hospital, Sichuan, China, and written informed consent was obtained from all subjects. Blood samples were collected from patients with Kawasaki disease (*n* = 19) at Chengdu Women’s and Children’s Central Hospital. The diagnosis of Kawasaki disease follows the criteria proposed by the Kawasaki Disease Research Institute in Japan, and patients who received anticoagulation, antiplatelet or hormone therapy were excluded from the study. Blood samples from children who had physical examinations were considered as healthy controls (*n* = 19) and were matched for age and sex. In another study, patients with Kawasaki disease (*n* = 57) were recruited from Chengdu Women’s and Children’s Central Hospital, and they were randomly divided into two groups: one group received conventional treatment, including IVIG (2 g/kg/d, 1 d, iv) and aspirin (30–50 mg/kg/d, and 3–5 mg/kg/d after the fever) (*n* = 30, average age of 2.6-year), and the other group received conventional treatment together with tanshinone IIA (1 mg/kg/d, 5–7 d, iv) (*n* = 27, average age of 2.3-year). The blood samples were collected 1–2 h before treatment and 8–12 h after treatment. The peripheral blood mononuclear cells (PBMCs) were isolated using Ficoll and cultured in Roswell Park Memorial Institute (RPMI) 1640 medium containing 10% fetal bovine serum (FBS); they were randomly treated with tanshinone IIA (1, 3, or 10 mg/L) or aspirin (5 mol/L) in the presence of phorbol 12-myristate 13-acetate (PMA) for 48 h.

### Rabbit Model of Immune Vasculitis

The animal care and all experimental procedures in the present study were approved by the Animal Research Ethics Committee of Chongqing Medical University, China. To induce immune vasculitis, the rabbits (3–4 weeks old, purchased from the animal center of Chongqing Medical University) were given two intravenous injections of 10% bovine serum albumin (BSA) (2.5 ml/kg) at 2-week interval (20). Age-matched rabbits given normal saline were regarded as control. Four days after the second dose of BSA, the rabbits were randomly treated with tanshinone IIA (5 mg/kg/d, 7 d, iv), aspirin [100 mg/kg/d, 7 d, ig (equals to 30–50 mg/kg/d of human)] or IVIG (2 g/kg, iv). The blood samples were collected while the rabbits were awake, and the rabbits were sacrificed by air embolism. Coronary arteries were isolated and fixed in 4% glutaraldehyde for 24 h followed by dehydration; then the coronary endothelial cells were observed under scanning electron microscope (S-3000N, Hitachi, Japan).

### Peripheral Blood Cell Counts

The rabbit peripheral blood was collected from the ear vein on day 7. The levels of platelets, white blood cells and hemoglobin were measured by the automated hematology analyzer (KX-21N, Sysmex, Japan) within 3 h after blood collection.

### Platelet Aggregation Assay

The blood samples of the rabbits were centrifuged at 120 × *g* for 10 min to obtain the platelet-rich plasma (PRP), and then further centrifuged at 800 × *g* for 15 min to obtain the platelet-poor plasma (PPP). The platelet count was adjusted to 100–600 × 10^9^/L before aggregation was determined by a multi-functional intelligence blood condense-meter (TYXN-96, China) in the presence of 2 μM adenosine diphosphate (ADP).

### Platelet Activation Assay

The activation of platelet was determined using Annexin V by FACSCalibur (Becton Dickinson, Franklin Lakes, NJ, United States). In brief, the PRP was resuspended in binding buffer at the concentration of 10^9^/L. Next, the suspended PRP (100 μl) was mixed with fluorescein-5-isothiocyanate (FITC)-Annexin V (5 μl), and the mixture was incubated at room temperature for 15 min in dark. The preparation was ready for the assay.

### Prothrombin Fragment 1 + 2 Assay

The concentration of prothrombin fragment 1 + 2 (F1 + 2) was determined by enzyme-linked immunosorbent assay (ELISA) specifically designed for rabbit. The assay was performed according to the protocol provided by the manufacturer. Briefly, standards and blood samples were incubated with biotin- and then horseradish peroxidase (HRP)-conjugated reagents. Unbound conjugates were removed by gentle washing. The chromogenic substrate 3,3′,5,5′-Tetramethylbenzidine (TMB) was used to quantify the HRP enzymatic reaction which converts the chromogen into yellow color after adding the stopping reagent. The intensity of yellow color developed, which was proportional to the concentration of F1 + 2, was measured as absorbance at the wavelength of 450 nm with a microplate spectrophotometer (Wellscan MK 3, Thermo, Waltham, MA, United States).

### Quantification of Serum Proinflammatory Cytokines

Serum levels of interleukin (IL)-1β, IL-6, IL-8, and tumor necrosis factor (TNF)-α were measured using respective ELISA kit according to the manufacturer’s instructions.

### Megakaryocyte Count in Bone Marrow

The bone marrow cells of the rabbits were collected from the femur immediately after they were sacrificed. The Wright’s staining was performed on the smear. The number of megakaryocytes was counted under microscope (Olympus CX31, Tokyo, Japan) within random field of views of 1.5 cm × 3.0 cm.

### Colony Forming Unit-Megakaryocyte Assay

The isolated bone marrow cells were cultured in Iscove’s Modified Dulbecco’s Medium (IMDM) with 10% FBS, 1% BSA, 7.8 μg/ml 2-mercaptoethanol, 0.34 mg/ml CaCl_2_, 50 ng/ml TPO, 100 U/ml penicillin, and 100 μg/ml streptomycin, and kept at 37°C with 5% CO_2_ for 12 days; for those isolated from BALB/c mice (male, 6–10 weeks old), tanshinone IIA was given *in vitro* at the doses of 3, 10, and 30 μg/ml. The cells were then fixed and stained with acetylcholinesterase (AChE). A Colony Forming Unit-Megakaryocyte (CFU-MK) was defined as a cluster of 3 or more AChE-positive cells (21).

### Colony Forming Unit-Fibroblast Assay

The isolated bone marrow cells were cultured in IMDM with 10% fetal calf serum (FCS), penicillin and streptomycin, at 37°C with 5% CO_2_ for 9 days followed by Giemsa staining. A Colony Forming Unit-Fibroblast (CFU-F) was defined as a cluster containing 20 or more fibroblasts (22).

### Human Megakaryocytic Cell Viability Assay

Human megakaryocytic cell line CHRF-288-11 was purchased from ATCC (Manassas, VA, United States); it was cultured in IMDM containing 10% FBS, 100 U/ml penicillin and 100 μg/ml streptomycin at 37°C with 5% CO_2_. The cells were seeded into a 6-well plate (1 × 10^5^ cells/ml) with 3% FBS in IMDM and incubated with or without tanshinone IIA (1, 3, 10, and 30 μg/ml) for 72 h followed by cell viability assay using trypan blue (0.4%).

### Annexin V-FITC/Propidium Iodide Apoptosis Assay

The Annexin V-FITC/propidium iodide (PI) apoptosis assay was conducted using FACSCalibur. Briefly, CHRF-288-11 cells were treated with or without tanshinone IIA (1, 3, and 10 μg/ml) for 72 h before being harvested. The cells were then suspended in the binding buffer at the density of 1 × 10^6^ cells/ml and incubated with Annexin V-FITC and PI at room temperature for 15 min in dark. At the end of incubation, the cells were washed, centrifuged and resuspended in the binding buffer for flow cytometric analysis.

### Mitochondrial Membrane Potential JC-1 Assay

Mitochondrial membrane potential was measured using JC-1 dye. In brief, CHRF-288-11 cells were treated with or without tanshinone IIA (10 μg/ml) for 72 h. The cells, at the density of 1 × 10^6^ cells/ml, were incubated with the freshly prepared working JC-1 solution at 37°C for 15 min. After that, the cells were washed and resuspended in the assay buffer for flow cytometric analysis.

### Active Caspase-3 Assay

Active caspase-3 assay was performed according to the instruction of the commercial assay kit. Briefly, CHRF-288-11 cells were treated with or without tanshinone IIA (10 μg/ml) for 72 h followed by incubation with the Cytofix/Cytoperm buffer for 20 min on ice. Then the cells were incubated with FITC-labeled caspase-3 antibody for 30 min in dark. The active caspase-3 was measured by FACSCalibur.

### Microarray Assay and Validation

The megakaryocytic CHRF-288-11 cells, treated without and with tanshinone IIA, were used to conduct the microarray assay with Affymetrix GeneChip human genome u133 plus 2.0 array (Affymetrix, Santa Clara, CA, United States). The results were analyzed with Affymetrix GeneChip Operating Software following the guidelines.^1^ The RNAs were extracted using RNA extraction kit (Ambion, Austin, TX, United States) according to the manufacturer’s instructions. Quantitative PCR was performed to validate the result of microarray assay.

### Statistical Analysis

The results are shown as means ± SEM with n referring to the number of animals used or cell passages. Statistical analysis was performed by using Student’s *t*-test (for two group comparisons) or ANOVA followed by the Bonferroni *post-hoc* test (for more than three group comparisons). The analysis was conducted by using Prism (version 8) (GraphPad Software, San Diego, CA, United States) and SPSS Statistics (version 25) (IBM, Armonk, NY, United States). Difference was considered to be statistically significant when the probability value (*P*) was less than 0.05.

### Materials

Acetylcholinesterase and phorbol 12-myristate 13-acetate were purchased from Sigma (St. Louis, MO, United States); FITC Annexin V assay kit, active Caspase-3 apoptosis kit and JC-1 were purchased from BD Biosciences (San Jose, CA, United States); IL-1β, IL-6, and TNF-α were purchased from MyBioSource (San Diego, CA, United States); IL-8 and P-selectin ELISA kits were purchased from ThermoFisher (Waltham, MA, United States); IMDM, RPMI1640, FBS, and BSA were purchased from Gibco (Waltham, MA, United States); tanshinone IIA was purchased from Shanghai Pharmaceuticals (Shanghai, China); TPO was purchased from PeproTech (Rocky Hill, NJ, United States).

## Results

### 1. The Effect of Tanshinone IIA on Patients With Kawasaki Disease

#### Tanshinone IIA Reduces Inflammatory Biomarkers and Platelet Activation in Kawasaki Disease Patients

**FIGURE 1:**
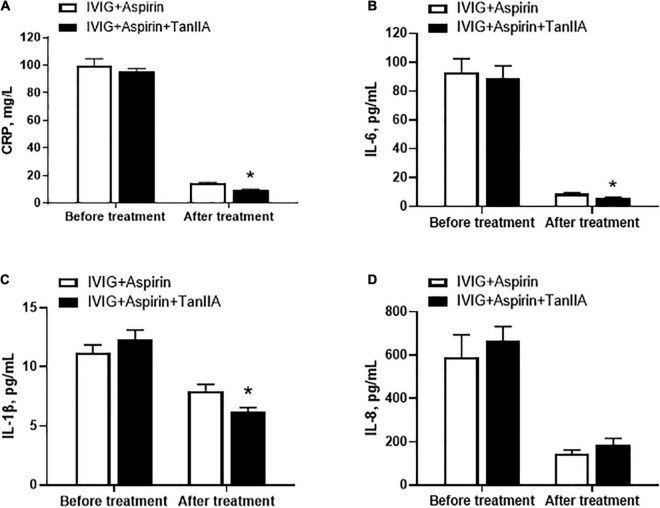
Tanshinone IIA lowers the serum levels of inflammatory biomarkers in Kawasaki disease (KD) patients. Tanshinone IIA (1 mg/kg/d, 5–7 d) was administrated intravenously to KD patients who were being treated with IVIG and aspirin. The serum levels of **(A)** CRP, **(B)** IL-1β, **(C)** IL-6, and **(D)** IL-8 were measured by ELISA. Data are shown as means ± SEM. IVIG + aspirin: *n* = 30; IVIG + aspirin + TanIIA: *n* = 27. *P < 0.05 *versus* IVIG + Aspirin (After treatment). IVIG, intravenous immunoglobulin; KD, Kawasaki disease; TanIIA, tanshinone IIA.

In order to examine the therapeutic effect of tanshinone IIA, KD patients were administrated with tanshinone IIA (1 mg/kg/d) for 5–7 days in addition to conventional treatment of IVIG (2 g/kg/d) and aspirin (30–50 mg/kg/d). No side effects such as bleeding, allergy or rash were observed in patients with tanshinone IIA prescription. Before the treatment, the serum levels of C-reactive protein (CRP), IL-1β, IL-6, and IL-8 were comparable between groups (Figure 1). Conventional treatment lowered the concentrations of these inflammatory biomarkers, and tanshinone IIA further reduced them significantly, except IL-8 (Figure 1), suggesting that tanshinone IIA has anti-inflammatory effect in KD patients.

**FIGURE 2:**
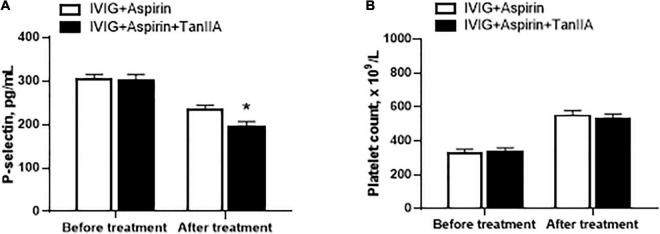
Effect of tanshinone IIA on serum level of P-selectin and platelet counts in KD patients. Tanshinone IIA (1 mg/kg/d, 5–7 d) was administrated intravenously to KD patients who were being treated with IVIG and aspirin. **(A)** The serum level of P-selectin was measured by ELISA and **(B)** the platelet counts were obtained from blood test. Data are shown as means ± SEM. IVIG + aspirin: *n* = 30; IVIG + aspirin + TanIIA: *n* = 27. *P < 0.05 *versus* IVIG + Aspirin (After treatment). IVIG, intravenous immunoglobulin; KD, Kawasaki disease; TanIIA, tanshinone IIA.

In addition to elevated levels of proinflammatory biomarkers, abnormal platelet function and number are also the typical features of KD patients. P-selectin is regarded as the marker of platelet activation. Tanshinone IIA treatment (1 mg/kg/d, 5–7 d) significantly reduced serum P-selectin level of KD patients, the effect of which was more promising when compared to that of conventional treatment (Figure 2A). However, tanshinone IIA did not further alter the platelet count in KD patients who were undergoing conventional treatment (Figure 2B).

#### Tanshinone IIA Suppresses the Secretion of Proinflammatory Cytokines/Chemokines in Peripheral Blood Mononuclear Cell of Kawasaki Disease Patients

**FIGURE 3:**
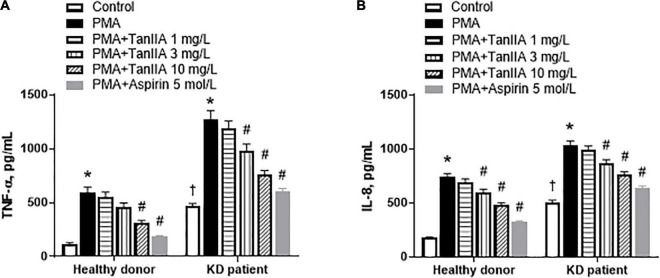
Tanshinone IIA lowers the levels of TNF-α and IL-8 in PBMC of KD patients and healthy controls in the presence of PMA. The isolated PBMCs were treated with tanshinone IIA (1, 3, or 10 mg/L) or aspirin (5 mol/L) in the presence of PMA for 48 h. The levels of **(A)** TNF-α and **(B)** IL-8 were measured by ELISA. Data are shown as means ± SEM. *n* = 19. ^†^*P* < 0.05 *versus* Healthy donor; *P < 0.05 *versus* respective Control; ^#^*P* < 0.05 *versus* respective PMA. PBMC, peripheral blood mononuclear cells; KD, Kawasaki disease; PMA, Phorbol 12-myristate 13-acetate; TanIIA, tanshinone IIA.

To investigate the effect of tanshinone IIA on the secretion of proinflammatory cytokines/chemokines *in vitro*, the PBMCs of KD patients and healthy children were isolated. The secretions of proinflammatory cytokine TNF-α and chemokine IL-8 in PBMC from KD patients were significantly higher than those from healthy donors, and were further augmented by PMA in both groups (Figure 3). In the presence of PMA, tanshinone IIA lowered the levels of TNF-α and IL-8 in a dose-dependent manner; these reductions were more obvious at the concentration of 3 or 10 mg/L (Figure 3). Aspirin also significantly reduced the concentrations of these cytokines (Figure 3). The results suggest that tanshinone IIA, in line with aspirin, is able to suppress PMA-induced inflammation in PBMC of KD patients.

### 2. The Effect of Tanshinone IIA on Rabbits With Immune Vasculitis

#### Tanshinone IIA Alleviates Coronary Endothelial Damage in Rabbits With Immune Vasculitis

**FIGURE 4:**
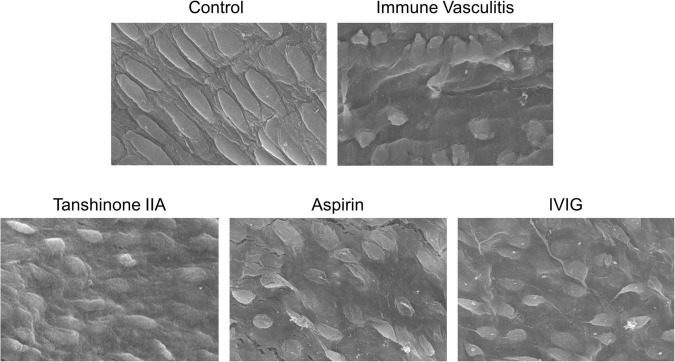
Representative images of morphological changes of rabbit coronary endothelial cells. The rabbits with immune vasculitis were randomly treated with tanshinone IIA (5 mg/kg/d, 7 d, iv), aspirin (100 mg/kg/d, 7 d, ig) or IVIG (2 g/kg, iv); the coronary endothelial cells of rabbits in different experimental groups were observed by scanning electron microscope at 1000 times magnification.

Weanling rabbits with BSA-induced immune vasculitis were used to mimic the pathology of KD. The image of rabbit coronary artery in control group showed complete and normal endothelial cells, however, the coronary endothelial cells of rabbits with immune vasculitis became disordered and swelling, which led to progressive necrosis (Figure 4); such loss of structural integrity and endothelial dysfunction suggest that the animal model of human KD was successfully established (20, 23). In tanshinone IIA, aspirin and IVIG groups, those endothelial damages were alleviated and the cells were back into shape (Figure 4), suggesting that tanshinone IIA attenuated coronary artery lesions in rabbits with immune vasculitis.

#### Tanshinone IIA Reduces Platelet Count and Functions in Rabbits With Immune Vasculitis

The blood cell counts and hemoglobin level were examined in rabbits with immune vasculitis and their healthy counterparts. In immune vasculitis rabbits, the number of platelets and white blood cells were significantly higher compared to their healthy counterparts; however, the level of hemoglobin decreased (Table 1). Tanshinone IIA (5 mg/kg/d, 7 d) statistically reduced the platelet count, but it did not affect the white blood cell count and hemoglobin level (Table 1).

**TABLE 1:** Blood cell counts and hemoglobin level in rabbits with immune vasculitis.

Group	PLT (× 10^9^/L)	WBC (× 10^9^/L)	Hb (g/L)
Control	690.40 ± 23.45	7.62 ± 0.47	140.00 ± 7.51
Immune Vasculitis	986.00 ± 98.62 *	15.67 ± 2.75 *	117.00 ± 5.93 *
Tanshinone IIA	557.60 ± 35.13 ^#^	13.83 ± 1.37	118.00 ± 3.31

Data are shown as means ± SEM. n = 5–6. *P < 0.05 versus Control; ^#^P < 0.05 versus Immune Vasculitis. PLT: platelet; WBC: white blood cell; Hb: hemoglobin.

**FIGURE 5:**
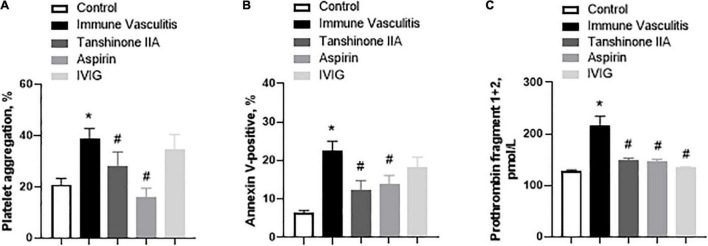
Tanshinone IIA reduces the platelet functions in rabbits with immune vasculitis. The rabbits with immune vasculitis were randomly treated with tanshinone IIA (5 mg/kg/d, 7 d, iv), aspirin (100 mg/kg/d, 7 d, ig) or IVIG (2 g/kg, iv); and then **(A)** platelet aggregation, **(B)** platelet activation and **(C)** prothrombin fragment 1 + 2 assays were conducted. Data are shown as means ± SEM. *n* = 6. *P < 0.05 *versus* respective Control; ^#^*P* < 0.05 *versus* respective Immune Vasculitis. IVIG, intravenous immunoglobulin.

The platelet aggregation, activation and prothrombin fragment 1 + 2 (F1 + 2) in rabbits with immune vasculitis were significantly augmented when compared to their healthy counterparts; tanshinone IIA (5 mg/kg/d, 7 d) statistically inhibited those parameters and such inhibitory effects were comparative to those induced by Aspirin (100 mg/kg/d, 7 d) (Figure 5). IVIG (2 g/kg) significantly inhibited F1 + 2 in rabbits with immune vasculitis (Figure 5C); however, it did not affect the platelet aggregation and activation (Figures 5A,B).

#### Tanshinone IIA Decreases the Serum Levels of Proinflammatory Cytokines in Rabbits With Immune Vasculitis

**FIGURE 6:**
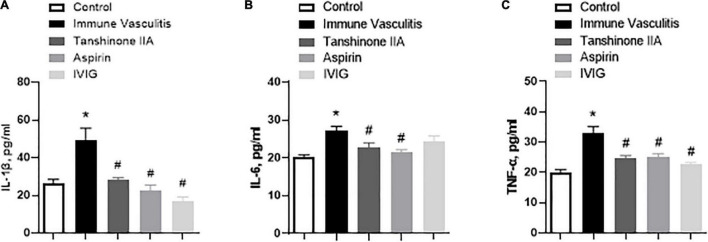
Tanshinone IIA decreases the serum levels of **(A)** IL-1β, **(B)** IL-6, and **(C)** TNF-α in rabbits with immune vasculitis. The rabbits with immune vasculitis were randomly treated with tanshinone IIA (5 mg/kg/d, 7 d, iv), aspirin (100 mg/kg/d, 7 d, ig), or IVIG (2 g/kg, iv). The concentrations of the proinflammatory cytokines were measured by respective commercial ELISA kit. Data are shown as means ± SEM. *n* = 6. *P < 0.05 *versus* respective Control; ^#^*P* < 0.05 *versus* respective Immune Vasculitis. IVIG: intravenous immunoglobulin.

In line with the findings of clinical studies on KD patients, the serum levels of proinflammatory cytokines IL-1β, IL-6, and TNF-α were significantly higher in rabbits with immune vasculitis than in healthy controls (Figure 6), which were statistically reduced by tanshinone IIA (5 mg/kg/d, 7 d), aspirin (100 mg/kg/d, 7 d) or IVIG (2 g/kg; except its inhibitory effect on serum IL-6 level) (Figure 6). In addition, the IL-1β level was positively correlated with platelet count and aggregation (*r* = 0.55, *p* = 0.012 and *r* = 0.603, *p* = 0.03, respectively).

#### The Anti-megakaryocyte Effect of Tanshinone IIA in Rabbits With Immune Vasculitis

**FIGURE 7:**
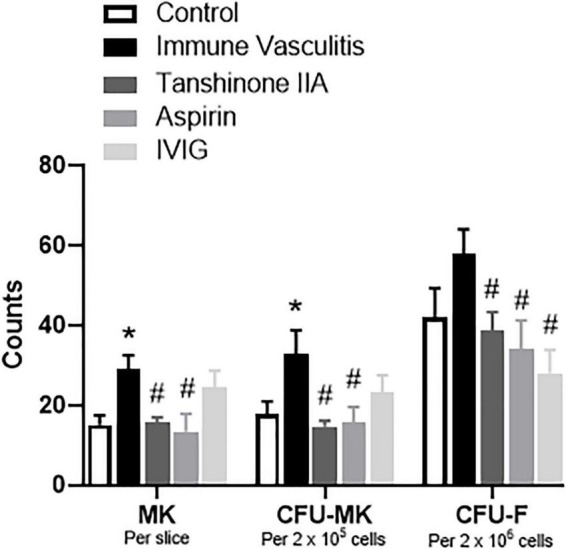
Tanshinone IIA reduces the number of megakaryocytes and the formations of CFU-MK and CFU-F in rabbits with immune vasculitis. The rabbits with immune vasculitis were randomly treated with tanshinone IIA (5 mg/kg/d, 7 d, iv), aspirin (100 mg/kg/d, 7 d, ig), or IVIG (2 g/kg, iv). The bone marrow cells were collected from the femur; the isolated cells were then cultured in IMDM with specific growth factors. Data are shown as means ± SEM. *n* = 6. *P < 0.05 *versus* respective Control; ^#^*P* < 0.05 *versus* respective Immune Vasculitis. IVIG, intravenous immunoglobulin; MK, megakaryocyte; CFU-MK, colony-forming unit-megakaryocyte; CFU-F, colony-forming unit-fibroblast.

To determine the role of tanshinone IIA in megakaryopoiesis, its effects on megakaryocyte counts within bone marrow, and the formation of CFU-MK and CFU-F *in vitro* were examined in rabbits with immune vasculitis. The number of bone marrow megakaryocytes and the formation of CFU-MK *in vitro* were significantly augmented in immune vasculitis than in healthy controls, while the increase in CFU-F formation was slight (Figure 7). Both tanshinone IIA (5 mg/kg/d, 7 d) and aspirin (100 mg/kg/d, 7 d) significantly reduced the megakaryocyte count and inhibited the formation of CFU-MK and CFU-F *in vitro*, to the levels comparable of control group (Figure 7), suggesting that tanshinone IIA inhibits megakaryopoiesis in rabbits with immune vasculitis.

### 3. The Effect of Tanshinone IIA on Mouse Primary Megakaryocytes

#### Tanshinone IIA Inhibits Mouse Colony Forming Unit-Megakaryocyte Formation *in vitro*

**FIGURE 8:**
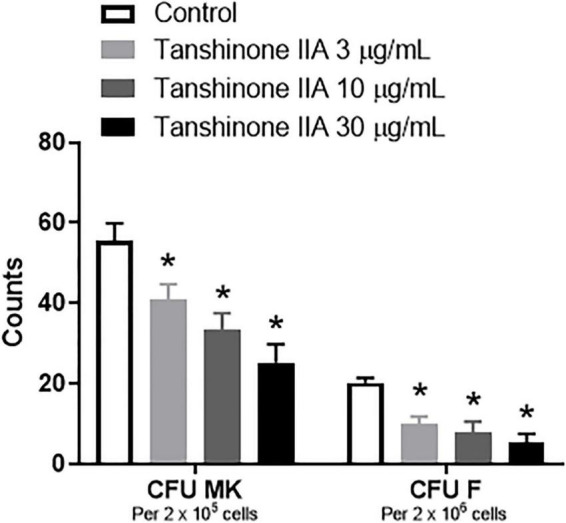
Tanshinone IIA inhibits the formation of mouse CFU-MK and CFU-F *in vitro*. The isolated bone marrow cells from BALB/c mice were cultured in IMDM with specific growth factors and then treated with different concentrations of tanshinone IIA (3, 10, and 30 μg/mL). Data are shown as means ± SEM. *n* = 3. * *P* < 0.05 *versus* respective Control. CFU-MK, colony-forming unit-megakaryocyte; CFU-F, colony-forming unit-fibroblast.

To investigate the effect of tanshinone IIA on CFU formation *in vitro*, BALB/c mice were used to collect bone marrow cells in which different concentrations of tanshinone IIA were incubated. Tanshinone IIA (3, 10 and 30 μg/mL) caused inhibition in the formation of CFU-MK and CFU-F in a concentration-dependent manner, and such inhibitions were statistically significant (Figure 8), which was in line with the findings *in vivo* in rabbits with immune vasculitis.

### 4. The Effect of Tanshinone IIA on Human Megakaryocytic CHRF-288-11 Cells

#### Tanshinone IIA Induces Apoptosis of Megakaryocytic CHRF-288-11 Cells

**FIGURE 9:**
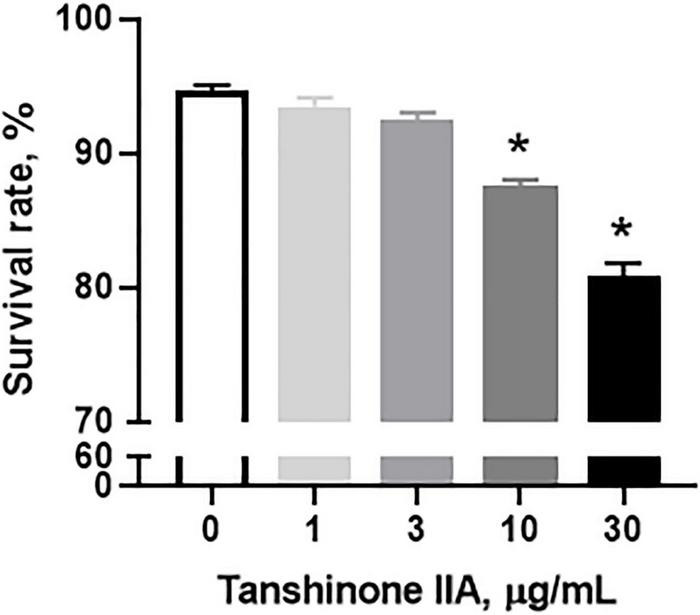
Tanshinone IIA induces a dose-dependent reduction in CHRF-288-11 cell viability. CHRF-288-11 cells were treated with different concentrations of tanshinone IIA (1, 3, 10, and 30 μg/mL) for 72 h. Data are shown as means ± SEM. *n* = 3. *P < 0.05 *versus* Tanshinone IIA 0 μg/mL.

**FIGURE 10:**
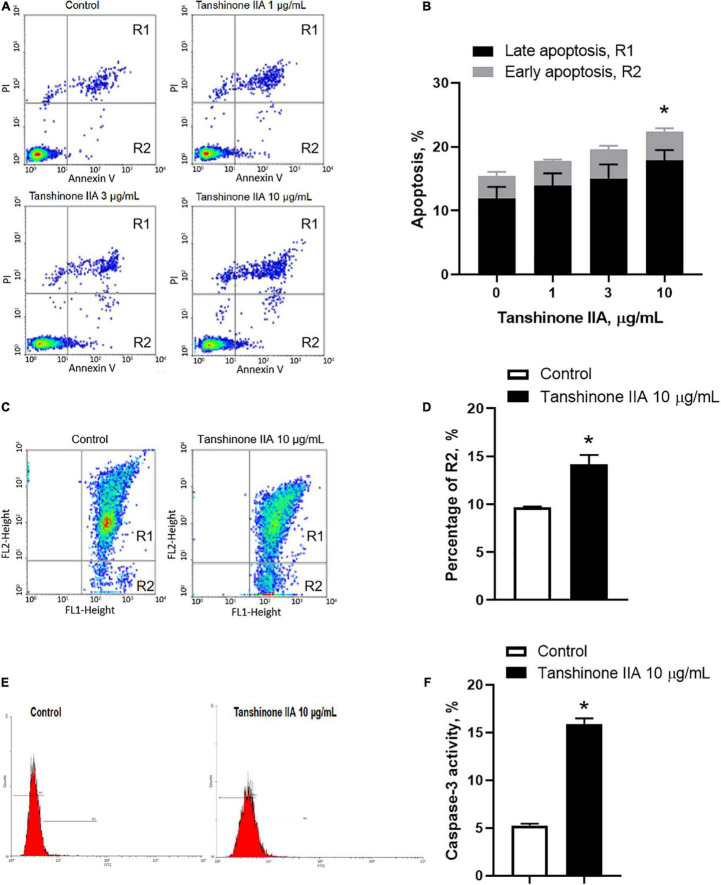
Tanshinone IIA induces apoptosis of CHRF-288-11 cells. Cells were treated without (Control) or with different concentrations of tanshinone IIA (1, 3, and 10 μg/mL) for 72 h before being subjected to **(A,B)** Annexin V/PI staining, **(C,D)** mitochondrial membrane potential JC-1 assay and **(E,F)** caspase-3 activity assay. Data are expressed as **(A,C,E)** representative flow cytometry and **(B,D,F)** shown as means ± SEM. *n* = 3–4. *P < 0.05 *versus* Control.

Tanshinone IIA (1, 3, 10, and 30 μg/mL) induced a dose-dependent reduction in the viability of CHRF-288-11 cells; the cell survival rate dropped significantly at 10 and 30 μg/ml (Figure 9). In order to examine the role of tanshinone IIA in megakaryocyte apoptosis, Annexin V/PI, mitochondrial membrane potential and Caspase-3 activity assays were conducted. The number of apoptotic cells increased as the concentration of tanshinone IIA went higher; at the concentration of 10 μg/mL, tanshinone IIA statistically increased the number of late apoptotic and necrotic cells (R1, Annexin V + /PI +) (Figures 10A,B). However, early apoptotic cells were not significantly affected by tanshinone IIA treatment. In addition, tanshinone IIA (10 μg/mL) significantly increased the proportion of CHRF-288-11 cells containing JC-1 monomers (R2), which had lower mitochondrial membrane potential indicating apoptosis (Figures 10C,D). The above results were verified by Caspase-3 activity assay which showed that tanshinone IIA (10 μg/mL) indeed augmented the activation of Caspase-3 (Figures 10E,F), suggesting that tanshinone IIA induces apoptosis of megakaryocytes.

#### Tanshinone IIA Induces Apoptosis Through Up-Regulating TNFRSF9 and RIPK1

**FIGURE 11:**
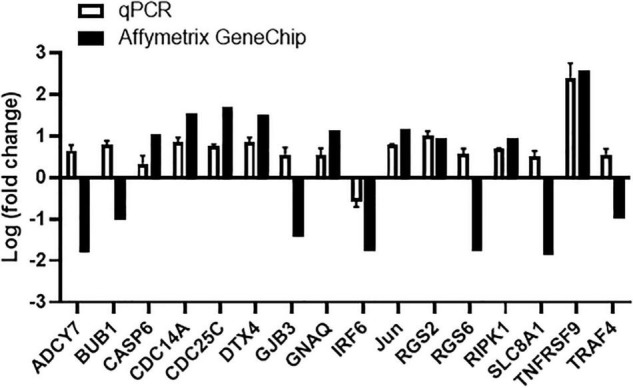
Tanshinone IIA regulates mRNA expression of genes involved in apoptosis-related signals in CHRF-288-11 cells, including up-regulating TNFRSF9. Quantitative PCR and Affymetrix GeneChip were performed to detect the gene expressions before and after Tanshinone IIA treatment. Data are shown as means ± SEM. *n* = 3.

In order to examine the molecular mechanisms involved in tanshinone IIA-induced apoptosis, human megakaryocytic CHRF-288-11 cells were subjected to microarray assay with Affymetrix GeneChip to identify the genes that were differently expressed after tanshinone IIA treatment. Several groups of genes have been identified to be up- or down-regulated in tanshinone IIA-treated CHRF-288-11 cells, including those involved in apoptosis, calcium regulation and cell cycle checkpoints. Genes that had log_2_ (fold change) greater than 1 or less than −1 after tanshinone IIA treatment were selected, and then were validated by q-PCR. Among apoptosis-related genes, the most significantly upregulated one was TNF receptor superfamily member 9 (TNFRSF9) with log2 (fold change) approximates to 2.50, corresponding to around 6-fold up-regulation (Figure 11). In addition, the receptor (TNFRSF)-interacting serine/threonine-protein kinase 1 (RIPK1), a protein that likely interacts with TNFRSF9, was also up-regulated after tanshinone IIA treatment to around 2 folds (Figure 11). The results suggest that TNFRSF9 and RIPK1 might be involved in the signaling pathway of tanshinone IIA-induced apoptosis.

## Discussion

Kawasaki disease is a febrile disease in children with immune vasculitis as the major pathological change. Since around 20% patients are resistant to the conventional treatment of IVIG or aspirin, potential alternative medication with better compliance is needed. Tanshinone IIA is the active and major lipid-soluble component of Danshen, which has been used for centuries to treat cardiovascular diseases. The present study demonstrated that tanshinone IIA may be a promising alternative therapy for the treatment of KD with better anti-inflammatory and anti-platelet effects than the combination of aspirin and IVIG; the underlying mechanisms may include tanshinone IIA’s inhibition in IL-mediated megakaryopoiesis and the induction of TNFRSF9/RIPK1/caspase-dependent megakaryocytic apoptosis.

The present study used rabbits with immune vasculitis as the animal model of human KD (20, 23, 24) to examine the effect of tanshinone IIA on inflammatory responses and especially, the megakaryocyte-platelet axis. In general, immune vasculitis could be established through intravenous injection of heterogeneous proteins; in this study, BSA was used to induce immune vasculitis in weanling rabbits. After two intravenous injections of BSA, the coronary endothelial cells of the rabbits became swelling and necrotic, and infiltration of inflammatory cells and rupture of elastic fibers could be well observed. In addition, the serum levels of proinflammatory cytokines, such as IL-1β, IL-6, and TNF-α, were also up-regulated, all of which suggest that the immune vasculitis model is successfully established.

Increasing evidences point to the extensive crosstalk between inflammation and coagulation, which leads to a variety of disorders affecting the cardiovascular system (25). During the inflammatory state, resting endothelial and blood cells become activated and start to express different tissue factors, which are the primary initiators of blood coagulation cascade that eventually activate protease factor X to produce thrombin (26). Thrombin serves as the strong stimulus for the production of platelets. In turn, coagulation also considerably modulates inflammatory activity. Factor X, thrombin and fibrin can activate endothelial cells and induce the synthesis of IL-6 and IL-8; clotting blood *in vitro* produces IL-1β and IL-8 as well (27). The present study indeed shows that the releases of proinflammatory cytokines in rabbits with immune vasculitis and in PBMCs of KD patients were higher than those in their respective healthy controls; the platelet count and functions in rabbits with immune vasculitis were also increased. The serum level of IL-1β was positively correlated with platelet count and aggregation, which confirms that inflammation and coagulation are complementary. In rabbits with immune vasculitis, the platelet count, platelet activation and aggregation, and prothrombin fragment 1 + 2, which is produced during the hydrolysis of Factor X complex, were increased compared to their healthy counterparts; tanshinone IIA reduces all these parameters, and such beneficial effect is thought to be associated with augmentation of prostacyclin and reduction in thromboxane, both of which lead to increase in cAMP within the platelet and thus preventing the aggregation (28, 29). Aspirin inhibits platelet aggregation *via* reducing cyclooxygenase (COX)-1, and the inhibitory effect of aspirin on COX-1 is greater than that on COX-2, therefore, aspirin resistance usually occurs in patients with high level of inducible COX-2, which increases the production of prostaglandin H_2_ and thus of thromboxane A_2_, leading to platelet aggregation. Numerous research studies have demonstrated that tanshinone IIA reduces COX-2 expression in inflammatory status (30–33), and thus in KD patients and animals with vasculitis, tanshinone IIA may inhibit platelet aggregation *via* reducing COX-2 level to treat aspirin resistance.

In addition, tanshinone IIA lowered the levels of IL-1β, IL-6, TNF-α, or IL-8 in rabbits with immune vasculitis and in PBMCs of KD patients, suggesting that the anti-inflammation effect of tanshinone IIA might contribute to its potential therapeutic role in KD. According to the literature, tanshinone IIA and aspirin may share the same mechanisms in inhibiting inflammatory responses, which through the prevention of dendritic cell maturation, leading to decreased T cell activation and thus less inflammatory cytokine release (34). Given that tanshinone IIA does not have the side effect of gastric mucosal damage or gastrorrhagia as aspirin does, it may be an ideal alternative therapy in terms of anti-inflammation and anti-platelet effects in treating KD.

Megakaryocyte is the platelet-producing cell. Several inflammatory cytokines are involved in the regulation of megakaryopoiesis and thus contributing to the pathogenesis of thrombocytosis in vasculitis. The inflammatory cytokines IL-1β and IL-6, together with TPO, are the essential growth factors for MK and platelets (17–19). The elevated inflammatory cytokines in immune vasculitis may contribute to the increase of MK and platelet. In consideration of the inhibitory effect of tanshinone IIA on inflammation and platelet, the undefined role of tanshinone IIA in megakaryopoiesis was further examined. In rabbits with immune vasculitis, the MK count and formation of CFU-MK were indeed higher than those in healthy controls. Tanshinone IIA, both *in vivo* and *in vitro*, inhibited the growth of MK, therefore, it is reasonable to speculate that the anti-platelet effect of tanshinone IIA may be mediated *via* inhibiting IL-induced megakaryopoiesis.

Bone marrow stromal cells play an important role in supporting the HSC/HPCs within the microenvironment; they release different cytokines, including stem cell factor (SCF), TPO, IL-6, and IL-11, to promote the proliferation and differentiation of HSC/HPCs (35). The present study shows that tanshinone IIA reduced the formation of CFU-F, which may also contribute to its anti-megakaryocyte and thus the anti-platelet effects.

In addition to the inhibition in megakaryopoiesis, the effect of tanshinone IIA on megakaryocytic apoptosis was also examined. The present study used megakaryocytic CHRF-288-11 cell lines, and found out that tanshinone IIA dose-dependently increased the number of annexin V-positive cells; fluorescent annexin V detects the anionic phospholipid phosphatidylserine located in the outer leaflet during cell apoptosis, suggesting that tanshinone IIA induces apoptosis of megakaryocytic cells. Mitochondrial dysfunction is regarded to be central to the apoptotic pathway; the opening of mitochondrial permeability transition pore triggers depolarization of the transmembrane potential and the release of apoptogenic factors into the cytosol, such as cytochrome c, which in turn activates caspase-9, and then activates caspase-3 and caspase-7, finally resulting in cell death (36). Tanshinone IIA reduced the polarized mitochondrial membrane potential of CHRF-288-11 cells and increased the activation of caspase-3, suggesting that tanshinone IIA induces apoptosis through an intrinsic mitochondrial pathway in a caspase-dependent manner.

To further determine the molecular mechanisms involved in the pro-apoptosis effect, microarray was conducted to identify the genes that were differently expressed after tanshinone IIA treatment in megakaryocytic CHRF-288-11 cells. Several groups of genes that implicated in apoptosis, calcium regulation and cell cycle checkpoints were found and were validated using quantitative PCR. The most significantly up-regulated gene after tanshinone IIA treatment was TNF receptor superfamily member 9 (TNFRSF9, also known as CD137), which is primarily involved in apoptosis and inflammation (37, 38). Research studies have shown that TNF-α, the receptor of which is a homologous to TNFRSF9, is able to induce two distinct caspase-8 activation pathways (39). In addition, receptor (TNFRSF9)-interacting protein kinase 1 (RIPK1), which can form RIPK1/Fas-associated death domain (FADD)/Caspase-8 complex is also up-regulated after tanshinone IIA treatment (39–41). The above information prompted us to speculate that tanshinone IIA-induced apoptosis in megakaryocytes may be partly mediated by the TNF-α-RIPK1-Caspase8-Caspase3 pathway. Certainly, this pathway does not exclude the possibilities of the involvement of other pathways that might induce cellular apoptosis. Taken together, tanshinone IIA, through inhibition in IL-induced megakaryopoiesis and TNFRSF9/RIPK1/caspase-dependent induction of megakaryocytic apoptosis, reduces the number of megakaryocytes and thus circulating platelets.

## Conclusion

In conclusion, the present study demonstrates that megakaryocytes are involved in the pathogenesis of immune vasculitis in a rabbit model. Tanshinone IIA attenuates immune vasculitis through inhibiting the formation of megakaryocytes and inducing megakaryocytic apoptosis, suggesting that tanshinone IIA may be an alternative therapy for the treatment of KD.

## Data Availability Statement

The original contributions presented in the study are included in the article/supplementary material, further inquiries can be directed to the corresponding authors.

## Ethics Statement

The studies involving human participants were reviewed and approved by the Chengdu Women’s and Children’s Central Hospital, China. Written informed consent to participate in this study was provided by the participants’ legal guardian/next of kin. The animal study was reviewed and approved by Chongqing Medical University, China.

## Author Contributions

HC, HS, XL, MZ, and MY designed the study. HC, HS, BL, HZ, LL, MZ, and MY performed the experiments and/or analyzed the data. WS, CL, WY, X-YZ, CC, XL, YY, MZ, and MY participated in the data interpretation. HC wrote the manuscript. BL, HZ, LL, X-YZ, MZ, and MY revised the manuscript. All authors contributed to the article and approved the submitted version.

## Conflict of Interest

The authors declare that the research was conducted in the absence of any commercial or financial relationships that could be construed as a potential conflict of interest.

## Publisher’s Note

All claims expressed in this article are solely those of the authors and do not necessarily represent those of their affiliated organizations, or those of the publisher, the editors and the reviewers. Any product that may be evaluated in this article, or claim that may be made by its manufacturer, is not guaranteed or endorsed by the publisher.

## Footnotes

1
http://www.affymetrix.com
